# Supplementary figures and images for: The Pancreatic Microbiome is Associated with Carcinogenesis and Worse Prognosis in Males and Smokers
Source: Cancers (Basel). 2020 Sep 18;12(9):2672. doi: 10.3390/cancers12092672 (PMC7565819; doi:10.3390/cancers12092672)

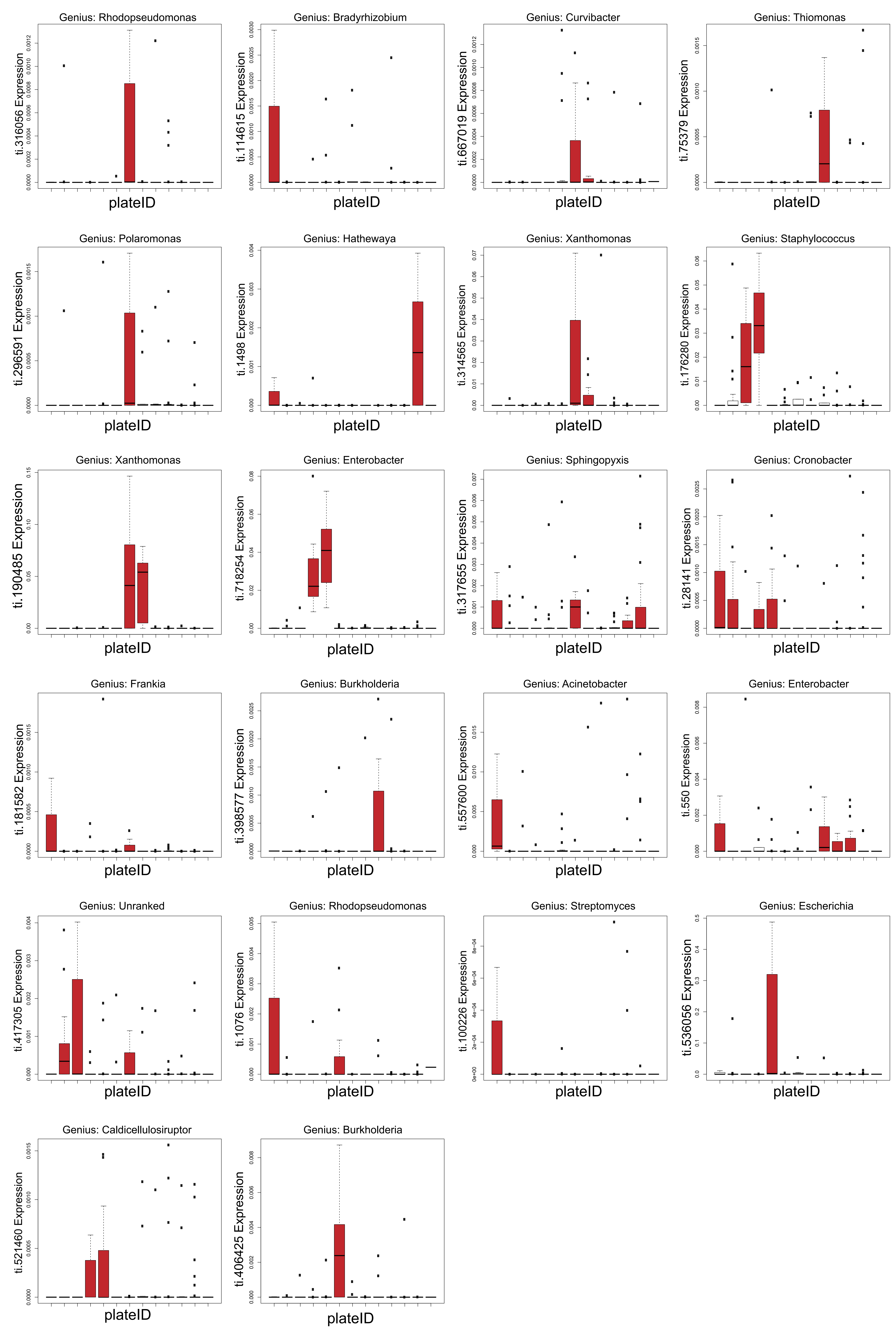

Supplement: Supplementary file 1 [file cancers-12-02672-s001.zip › Supplementary figure 1_Contamiantion correction by plate.pdf]

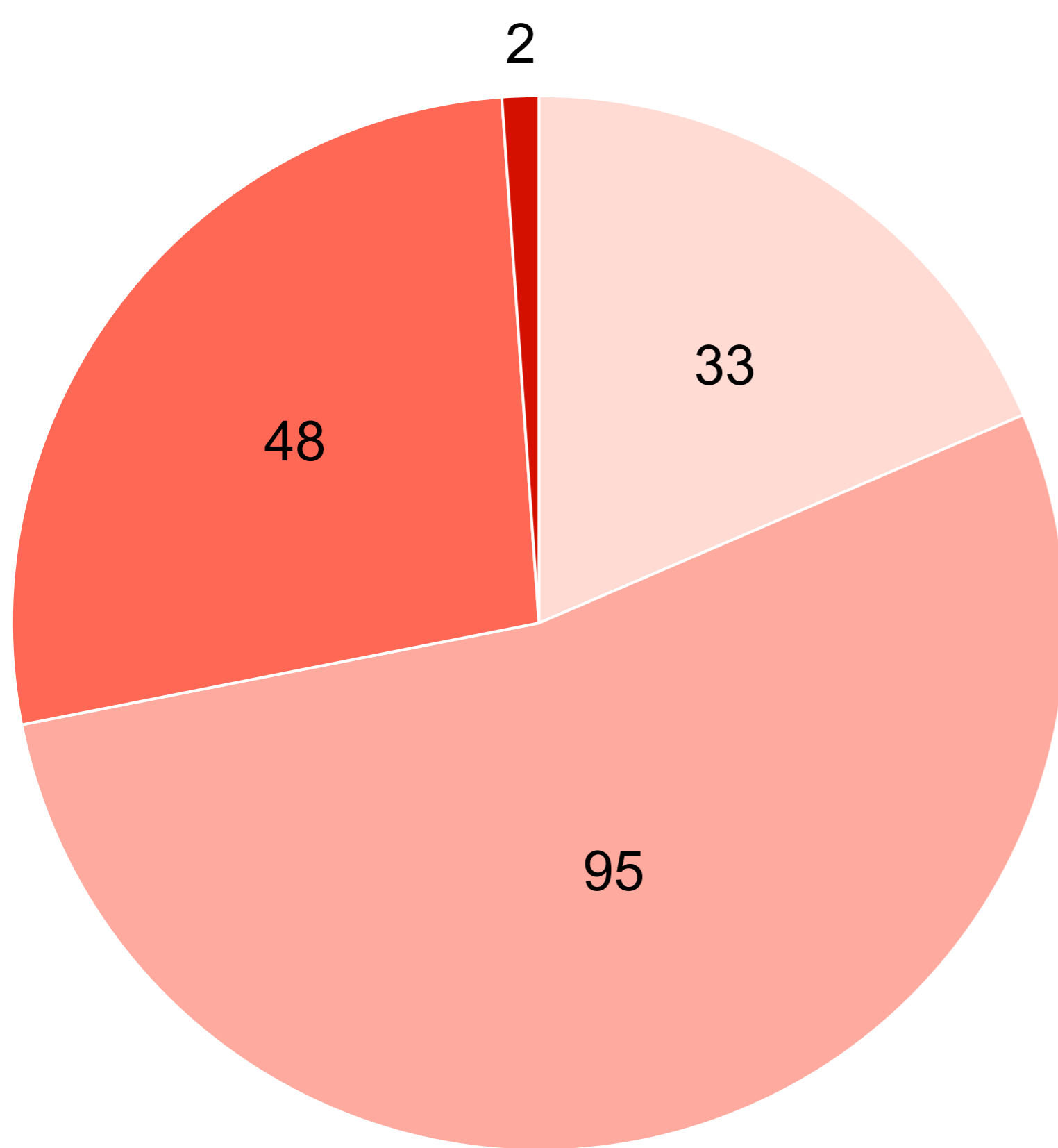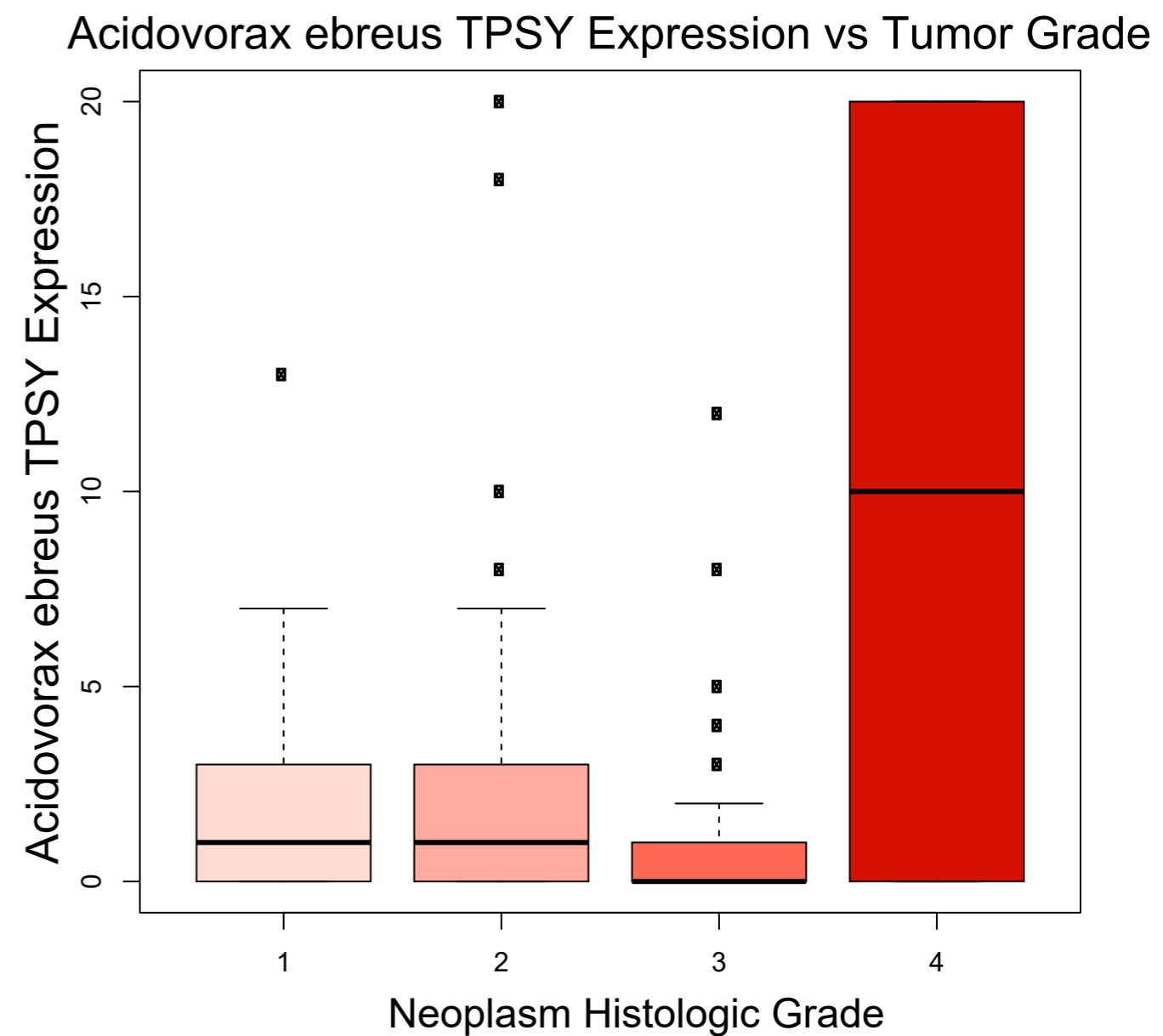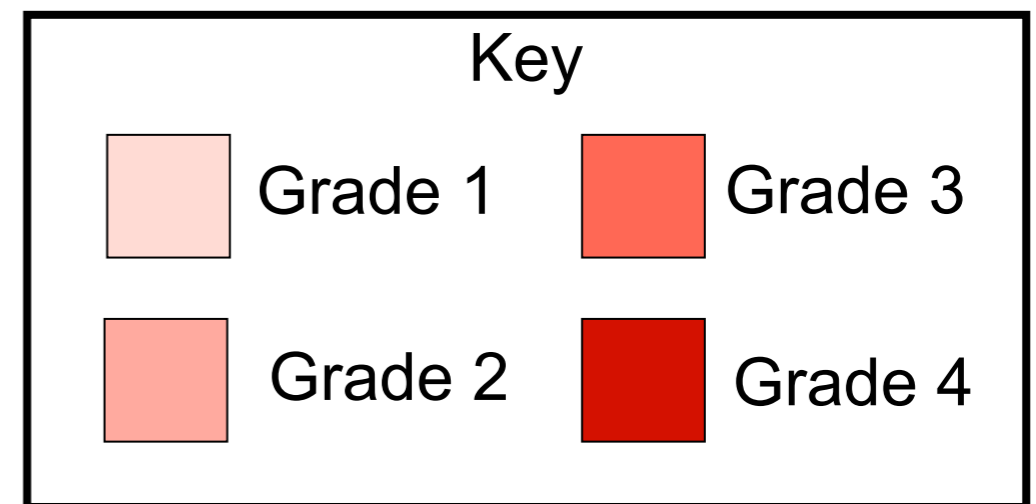

Supplement: Supplementary file 1 [file cancers-12-02672-s001.zip › Supplementary Figure 2_tumor grade.pdf]

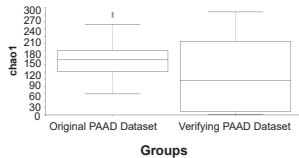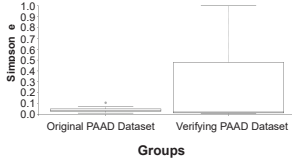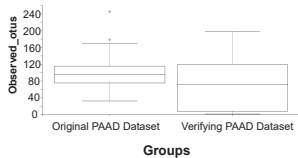

Supplement: Supplementary file 1 [file cancers-12-02672-s001.zip › Supplementary Figure 3_validation.pdf]
